# Medical and Philosophical Intelligence

**Published:** 1819-08

**Authors:** 


					[ 168 }
JWtSttal autt JMjilogopSfcal J-ttfclttgctue.
Kotiges of such of the TRANSACTIONS OF PHILOSOPHICAL societies, a at
RELATE TO MEDICINE AND ITS AUXILIARY SCIENCES.
J*OYAL SOCIETY of LONDON. Sitting of April 22d.?Si*
Eveuard Home read a paper on the ova of the opossum tribe. After
some allusions to his former paper on the formation of the ova in
quadrupeds, he observed, that the ova of the opossum tribe are formed
differently from those of quadrupeds, and eonstitnte two distinct
gradations between that class of animals and the ornithorhynchus
paradoxus; which last approaches so near the bird, as to complete the
series between quadrupeds and birds.
The formation of an ovum in the kangaroo, was first described.
This, when expelled from the corpus luteum, receives a yolk in the
fallopian tube, and subsequently an albumen in the uterus. The foe-
tus, when removed from the uterus into the marsupium, attaches itself
to the as formerly described. (Phil. Trans, vol. 85 and 100.)
In the wombat and the great and small opossum, instead of corpora
lutea, yolk-bags are imbedded in the substance of the ovarium ; and
there are two uteri with a fallopian tube to each, the ovum in each;
uterus being separately impregnated in its own cavity. The mode of.
formation Of the ova in the omythorhynchus paradoxus, constitutes,
the intermediate link between the opossum and bird. In this-auim&ly
the yolk-bags are imbedded in the ovaria ; and, instead of a regular
uterus, each fallopian tube swells out into a cavity, in which the ova
are impregnated.
A paper, on the case of a blue child, with the dissection, was read by
J. F. Wood, Esq. This child lived twenty-one months. On remov-
ing the pericardium after death, a large vein was observed, descending
on the left side of the thorax, and terminating in the right auricle of
the heart; in which the superior vena cava also terminated by a dis-
tinct opening. The auricle was large, and the foramen ovale pervious.
The aorta and pulmonary artery arose from the right ventricle, the ca-
vity of which was likewise large and strong, and had no communica-
tion with the left, except by a foramen through the septum which
divides the ventricles.
1Xoyat Society of Edinburgh.?At a late sitting of this society, a
letter was read from Dr. Alexander Kennedy to James Russell,
Esq. giving an account of the extraction of a worm from the aqueous
humour of a horse's eye, which was effected at Madras by Dr. William
Scot. Dr. Kennedy presented this worm to the society. It was found
by Captain Thomas Brown to be a new species of the ascaris, to
-which he gave the name of ascaris pellucidus. In a letter, dated
Madras, July 7th, 181S, which Dr. Kennedy has subsequently re-
ceived from Dr. Moore, who was present at the operation, is the fol-
lowing paragraph: " 1 received the worm, with the aqueous humour
of the eye, in a cup, and it continued to move with great vivacity for
some time, until I added a little w ater, when it instantly fell dead to
the bottom of the cup."
1
Medical and Phildsophital Intelligence. iGQ
Jiff. I ft, 4 paper by Or. FE rt<?tJso* was read, on the poisonous fishei
of the Cai ibbee Islands: The duthrrr endeavoured t? prove, (hat, in
kfl the larger fishes of prey, the poisonoos quality \tfas a rare and ac-
cidental occurrence ; and that it was found to be present only at a
certain season of the year, in one or two of the smalfer species Of fish,
Wofe particularly in the ycllotv-Mlled sprat, (the sardine dorS off (he
French, and ciupeathryssa of naturalists;) whence he inferred, that
the larger voracious fishes, such as the (raracosta, (pefca major of na-
turalists,) &c. became poisonous only at the times they hat) recently
been feeding on the smaller porsorious prey. The notioB of these be-
ing made poisonous from lying on copper-bants, or theft eating
the stinging blubbers, (themedmte and kohtkurice,) was refuted. Iti
Regard to the tests, it Was shown that none could be depended on ; that
nothing whatever conhl be discovered from inspection of the fish; that
the test of boiling a piece of silver with the suspected fish, proved no-
thing, whatever might be its actual quality ; that, so far from there*
being anymarks of disease in the viscera, or other parts, of poisonoas
fishes, tliey were generafiy found to be in the best state m every respect*
The poison of the yellow-billed'sprat was supposed to eiist iu thtf
animal at certain seasons of the year, and not to be occasioned by its
having fed on any undiscovered local marine poison, from the circumj
Stance of the otlier-smaller fishes of the same genus, that were found h!
the sajne places, never partaking of the poisonous nature; and from
the poison of the fish being more potent and deadly than any other
known, or even supposed, article of food eould be likely to oo<n4
municate.
With respect to remedies and antidotes, the efficacy of sugar was
alorte established as deserving of credit. Wines, spirits, and the con-
diments used at table, were belfeved to have obtained occasional credit
only from their being used in slight cases of.the poison, as would most
likely have terminated in a favourable manner without any remedy;
As a precaution in ail cases- of suspicious fish of the larger species^ the
cleaning them out as soon as caught-was recommended as a usefifl and
proper one, to prevent the carcase being further tainted by the lodg-
ment of any poisonous matter (such as that of the yellow-billed sprat)
recently swallowed; though it was sh-own, at the same time, that tlia
doing so, and even salting the fish afterwards, could not, in any i?w
stance, remove the poisonous impregnation so communicated to (head
voracious creatures, whose powers of assimilation, from the shortness
6f the Intestines and great size of the liver, must be supposed to be
infinitely quicker than what takes place amongst terrestrial animate*
It Was useful also in another way, by furnishing the material of the
only criterion hitherto discovered for detecting the poison, which was
shown, to be that of giving a portion of the liver or offal to some In*
ferior animal, (such as a cat, a duck, or a pig,) and ascertaining its
effects upon them before making use of the fish.
In our review of the first volume of the " Dublin Hospital Re-
ports/' we presented our readers with an abstract of the observations'
of frr. Colles respecting the connexion of disease of the utuBiikal
no. 246. z
170 Medical and Philosophical Intelligence.
vessels with trismus nascentium. Dr. Labatt, the master of the Lying-
in Hospital of Dublin, has recently published some cases of dissec-
tions,* which show that the former does not coincide with the latter so
frequently as the observations of Dr. Colles would indicate; and, from
the same affection of the umbilical vessels having been witnessed after
death from other diseases not attended with trismus, their relation as
cause and effect is rendered undetermined. We shall give an account
of histories of the cases related by Dr. Labatt.
" 1st Dissection.?The infant died of locked-jaw the eighth day
from its birth. With the exception of a slight appearance of ulcera-
tion, the umbilical fossa presented nothing peculiar. Its edges were
not raised, nor was its floor; a probe could not be introduced from
the fossa into the umbilical vein. On cutting into the abdomen, no
signs of inflammation or vascularity could be discovered in the perito-
neum covering the umbilical arteries or veins ; nor was there the
slightest appearance of a yellow watery fluid in the cellular substance
surrounding them. The vessels themselves, on being slit open, appeared
healthy, without^any thickening, marks of inflammation, or yellow
fluid, within their coats. No space occupied by a soft yellow sub-
stance could be detected on cutting into the umbilicus, from its internal
or peritoneal surface; nor did the extremities of the arteries or veins,
towards the navel, exhibit anything peculiar. They were all pervious
from within. Bladder was distended with urine."
" 2d Dissection.?This child died on the tenth day of a bowel-com-
plaint. We dissected it, in order to compare the appearances with
those remarked in the former subject; and we were struck with the
similarity they bore to certain parts of Mr. Colles'S description. The
fossa was raised in the centre into three papillae or small elevations,
which proved to be the orifices of the umbilical arteries and vein, en-
larged and covered with lymph. On cutting into the abdomen, the
peritoneum covering these vessels appeared more vascular than usual,
and the cellular substance surrounding them contained a yellow watery
fluid; their orifices remained open, and the coats were evidently thicker
than in the first case."
" 3d Dissection.?This subject was carried off by a diarrhoea on the
eighth or ninth day. No memorandum of the dissection was preserved ;
but I recollect there were some of those appearances which are said to
be confined to infants who had died of trismus.
" 4th Dissection.?Here also we neglected to preserve notes ; but,
though the infant died of locked-jaw, the umbilicus exhibited but few
of the appearances mentioned by Mr. Colles."
We regret that Dr. Labatt did not record the u few appearances
mentioned by Mr. Colles," which were witnessed in the last case; but,
in another instance,?
" 5th Dissection.?A fine plump boy: funis separated on the fifth
day; attacked with trismus on the same evening, and died in sixty
hours: skin forming the edge of fossa not more raised than natural*.
The central papilla, or raised part, was connected to the skin forming
the superior edge of the fossa, so that a probe could not be passed
round it. This papilla was occupied in its centre by the opening of
" * Edinburgh Medical and .Surg ical Journal, No. 59. ~ T~ . '
Medical and Philosophical Intelligence. 171
Hie umbilical rein, which was pervious; but there was not any ap-
pearance of ulceration round it. At the inferior edge of the fossa and
base of the papilla, the openings of the umbilical arteries were seen
surrounded with yellow matter, and the surface of this portion of the
fossa appeared ulcerated." No morbid appearances were witnessed in
the subsequent part of the examination.
In the 6th dissection, of a small female infant, in whom the funis
separated on the morning of the fourth day, and who was attacked
with trismus the same evening, and died in forty hours, the only ap-
pearance that could be considered morbid was "a yellow watery fluid,
covering and passing along the umbilical arteries. At the junction of
the vessels inside, corresponding to the fossa without, and under the
peritoneum, there was a thickening of the cellular substance, of a
yellowish appearance and pretty firm consistence."
The 7th dissection was of a child who died of trismus on the eighth
day, in whom none of the appearances described by Dr. Colles were
witnessed. The same remark is applicable to the 8th case.
In thepth dissection, of an infant who died on the fifth day, " of
an affection of the chest," the edges of the fossa were not elevated;
but its surface was covered with yeilow matter, and looked ulcerated.
The point within the abdomen corresponding to the fossa without,
was thickened, but not covered with yellow matter. On cutting into
i-t, however, this substance was evident, The extremity of the vein
was thickened.
The following case of success/id treatment of the cholera morbus of
India, was originally published in a Bengal newspaper. The writer
says: " I was requested to visit a bearer, who, three hours previously,
had been attacked with cholera morbus: there was incessant vomiting
and purging; his extremities were cold, as also the skin; the pulse
could not be distinguished. I, of course, entertained slight hopes of
his recovery, but gave him immediately forty grains of calomel, fol-
lowed by laudanum one hundred drops, twenty drops of oil of pepper-
mint in four ounces of brandy; and ordered the abdomen to be con-
stantly rubbed with warm brandy. The medicine remained on his
stomach for three-quarters of an hour, when the vomiting returned :
he vomited a worm of eight inches in length, but without any allevia.
tion of the sickness at the stomach. He had now eighty drops of
laudanum, twenty of oil of peppermint, and three ounccs of brandy,
as also twenty grains of calomel; after which he rapidly recovered,
without the slightest inconvenience from the calomel."
We extract the following observations concerning a young man born
deaf and blind, from a letter addressed to Professor Jameson, by Dr.
Hibbeut: they involve matters of much interest to naturalists in ge-
neral, as well as to physiologists.
u David Gilbert Tate is the name of this singularly-destitute being.
He is said to be 25 years of age. His parents are in very indigent cir-
cumstances. About two years previously to the birth of this youth,
tjiey had a daughter, who from her infancy displayed every symptom
55 2 -
172 Medical and Philo&dpkkat Intelligence.
ofidio&m. Thqre was that torpor of the mental faculties irhich is
denoted by an. indocility of apprehension, and an articulation which
could not be rendered subservient to the purposes of speech. Some
time after her birth, she became blind: she still lives^ and is 27 years
of age. It may be remarked, that the mother has had eight othor
children, who manifested none of the defects which, conjoined in one
individual, rendered his case so singular. On my visit to the miserable
hovel occupied by theTates, David Gilbert was warming himself by a
fire, in a posture not unlike that which is described as peculiar to the
Moors; that is, he was not actually seated, but seemed most at ease
with his chin occasionally resting upon his knees, whilst his extremities
weregathered up to the trunk. The sternum was much protruded-
The lumbar and dorsal vertebrae appeared to be somewhat curved;
but whether or not this was the effect of disease, or was a#i habitual
position of the trunk, which was bent forwards equally with the ster-
num, I could not learn. It was however a matter of grpat surprise to
find that this position was maintained in his gait, and to team from the
mother that no attempts ha<d bee# made to teach him to walk er,ect.
The parents of David had, from his birth, regarded Jam in the light ef
a forlorn creature, whose peculiarly bereft lot po tuition could amelio-
rate. To David Tate the erect attitude was certainly not habitual;
and, when induced by coercion, was maintained with yiery uneasy feel-
ings, whilst its continuance met with his most determined resisUncp.
When compelled to assume an erect position, an opportunity was off
fered of remarking his general physiognomy. IIis countenance cer-
tainly appeared very idiotic. Iiis forehead, which in the lower part
protruded, was in the upper part retreating; whilst the occiput ap-
peared rather flattened. His whole body seemed emaciated. His chi?
Was very prominent; his mouth remarkably wide; his nose very
sharp. The pupil of the eyes showed the pitchy-black and dilated ap-
pearance characteristic of amaurosis, and the iris did not contract or
dilate upon the sudden application or withdrawing of a caudle. When
first observed, David had no sensible object within his grasp : it was
then curious to observe the innumerable muscular contcactiuns of his
fingers, and the velocity with which their motions were executed, to
produce a rapid change of position ; showing, from a simple origin,
being neither more nor less than the solitary circumstance of varied
muscular contraction exerted in parts of the body best calculated to
producc the effect, that the enjoyments of this individual were derived.
On the mother being asked ii What did the boy like best to handle?"
she replied, " Each thing that he can alter the shape of." David's
intonations of voice, which I only heard when his painful feelings were
intended to be expressed at the erect position in which he was placed,
were somewhat remarkable: thoy were highly melodious, being uttered
rn almost every key. Previously to receiving food, his mother taps
his hand with a spoon, which is recognized by the poor object as a
signal that she is preparing to satisfy his hunger : in an instant, there-
tore, the hands of David are extended to receive the bason in which is
contained his pottage. The existence of memory, or of an association
of ideas, is, in the qext place, proved by the attachment which he is
said to express towards his mother, who constantly feeds him. This is
Medical and Philosophical Intelligence? 173
denoted by a restlessness whea he cannot, by feeling every object
around him, detect her presence: her maternal offices of kindness are
also preferred before those of any other individual."?Edinburgh
Philosophical Journal, No. i.
In connexion with such of the above observations as relate to the
hypotheses of Camper and of Gail, we may observe, that Professor
Blumenbach has recently added to his collection of crania, one of aa
ancient Greek, taken from a grave in Magna Grecia. Jit is particularly
distinguished by the gentle curve of the brow, and the perpendicular
position of the upper jaw. It may be considered as the prototype of
the ancient Grecian profile, and serves to show that the profiles la
Grecian works of art were not, as De Pau stated, merely " a style of
design adopted in some schools." The learned professor has also
added to his collection the skull of one of the Botecudos, a tribe of
cannibals who inhabit remote and retired districts in the interior of
Brazil: it is strikingly in contrast with that of the Greek already
mentioned, resembling more nearly, in its general aspect, that of the
orang-outang than even the most characteristic skull of the negro race.
Dk. John Davy, in his investigations of the properties of the urine
of several species of animals, has made some discoveries which are par-
ticularly interesting to physiologists, and which tend to show the cor*
rectness of the statements of M. Magendio respecting the influence of
animal food in the production of uric acid. Dr. Prout, some years
since, ascertained that the urine of the boa constrictor consisted en,
tirely of uric acid : Dr. Davy has now determined that the urine of
different species of serpents is of the same nature. When first evacu-
ated, it is of a butyrqeeous consistence, but becomes quite hard by ex-
posure to the air. It was always found to be uric agid, nearly in 3,
state of purity : this was also the case with the urine of lizards. That
of the alligator, besides uric acid, contains a large portion of carbonate
and phosphate of lime. The urine of turtles was a liquid containing
flakes of uric acid, and holding in solution a little mucus and common
salt, but no sensible portion of urea.
In the treatment of calculous complaints, when the concretions are
composed chiefly of uric acid, and in the form of sand or gravel, we
have had frequent occasion to remark of what considerable importance
it is that magnesia, (which is the best of the alkaline substances in the
generality of cases, since it can be taken freely without inconvenience,)
when exhibited in these affections, should be in a state of purity : pa-
tients, who have taken it for a great length of time in the state of a
carbonate without benefit, have experienced the most prompt relief
from the use of the pure earth.*
* We particularly recommend to our readers the magnesia of Mr. Lockyer,
of Bedford-street, Bedford-square. This chemist, by a new process, produces
tliat substance in a state of absolute puiity and in an impalpable powder, at *
very moderate expence ; but, we believe the utility of liis diseovery'is not g$n%
rally known to medical practitioners, frujji his not having taken the ordinary
means of introducing it to the notice of the public. We should add, that Mr.
Lflckyer is a commercial chemist ; and, therefore, that hi? magnesia |H4,y l>e
at his owtf manu&ctory.  "
[ 174 J
REPORT OF DISEASES.
AVERY careful and extensive enquiry amongst the medical practitioner* of
the metropolis, has rendered itapparent to us that the prevalence of typhous
fever has been lately much on the decline. In the cases which have come under
oiif own observation, this disease has been mild in its. character ; neither accom-
panied with very great excitement in its early stage, and, consequently, not
attended with much subsequent general depression of vitality. The most fre-
quent scats of local inflammation.have been the mucous membrane* of the lungs
and the brain : this affection in the latter organ, has more frequently followed than
preceded the pulmonic disease. It is a curious and interesting circumstance,
tiiat the mucous membrane of the respiratory organs is the most common seat of
local inflammation in fever during the summer season; whilst that of the gastric
system is chiefly affected in autumn and winter: this has been particularly no-
ticed by all those physicians who have followed the doctrines of Stalil, and have
consequently had the best opportunities for tracing the course of diseases uninter-
rupted by art, which they made their chief study. The writings of Sydenham,
Bfnrton} Duret, and Baillon, decisively point out the circumstance to which we
have alluded; as well as those of many late writers on fever, who have drawn their
descriptions from extensive experience. When local disease has not been se-
vere, and not affected a very important organ, the most favourable resuhs have
ensued from the use of such remedial measures as seem, to act by restoring the
balance of vital action ; and these have apparently been more prompt and more
fully beneficial than those which act by lessening the general vitality.
Some eases of intermittent fever have occurred to our observation, which, have
Veen developed after a sort of febricula without any well-marked local affection
had existed for a few days. We have had cause, in several instances, to witness
the propriety of reserve in the use of cinchona in the first stage of this form of
disease; for, when it has been thus employed, the fever has in some cases become
continued, and accompanied with evident and severe inflammation of the lungs
or brain, and sometimes of both; and this change in the character of the disease
we have been led to attribute to the cinchona.
Chronic inflammation of the peritoneum has lately been so frequent as to merit
notice amongst the prevalent diseases. From the want of acute or severe pain,
much fever, and any disturbance of the stomach and bowels, it is often treated
in a very ineffectual manner; and is sometimes suffered to run on to suppuration,
or serons effusion, i>efore the real nature of the affection is ascertained. We
have been called in consultation to a case, in an unmarried female, aged 19 years,
who had suffered slight pain and soreness about the abdomen for ten days before
medical advice was solicited. The apothecary who attended her had re-
course to blood-letting, laxatives, and calomel. Three days afterwards, when the
reporter first saw her, the navel had burst, and about four ounces of pus had
been evacuated. The patient is now convalescent.
Several cases of ascites have appeared, in the patients applying to public dis-
pensaries, apparently dependant on neglected peritoneal inflammation. The?e
have generally been successfully treated by purgatives and digitalis. The various
and important beneficial qualities of this drug are, we think, not sufficiently
known to practitioners in general. The judicious and observant Dumoulin,
after very long and extensive experience, said <k pressez-vous de faire usage d'un
remede qui fait de? merveilles depuis peu : il ne sera bientot bon a rienand
this is apparently applicable to digitalis.
Scarlet fever is rather prevalent, and has appeared lately in a more severe
form than that which it assumed at the period of our last report.
METEOROLOGICAL JOURNAL.
By Messrs. William Harris and Co. 50, Holborn, London.
From the 20th May to the 19th June, 1819, inclusive.
QS
May
20
21
22
23
24
25
26
27
28
29
30
31
Junf
1
2
3
4
5
6
7
8
9
10
11
12
13
14
15
16
17
18
19
c
o
Rai
gauge
,16
,75
,13
,U
,30
,15
,05
,16
,31
,09
,10
,20
,14
Therm.
57 65i52
56 65'49
55 67(51
57
64|52
61 53
50
47
45
45
5853
59,47
6555
65157
68'58
69'51
65^50
63*54
66154
62
63
65
60
57
55
59
6016855
6069!54
57,68*50
56164 52
5763
57 65
5967
67155
69:54
Bap.om.
29-59
29'52
29-73
29-71
29-91
29-93
29-86
29*87
29 90
29-92
30-20
30-09
S0-01
30-60
30-60
2950
S0-05
29-95
29-64
29-62
2965
29-74
30-00
30 02
30-07
30-00
29-79
29-95
29 89
30-00
SO'll
20.66
29-65
29-70
29-8S
29-96
29-91
29-93
29-83
29-90
30-00
30-10
so-ip
30*!?G
30*50
30-20
29-86
30-06
29 80
29*70
29 60
29-7C
29-91
S0-04
30-06
3002
29-91
29-83
29-85
3000
30-09
30-13
De Luc's
Hygkom.
Dry Damp
Wind.
SVV
ESE
8
SE
ESE
NE
E
ENE
ENE
NE
WNYV
W
W
SW
SW
W
WNVV
SW
SW
s
SW
WNW
W
SW
SW
NW
SW
NW
NE
NE
NE
N
SE
SE
S
ENE
ENE
ENE
NNE
E
NW
W
NW
SW
WSW
w
SSW
SW
SSE
SW
SSE
w
w
w va.
NE
WSW
SW
NNW
E
ENE
N
N
Atmospheric
Variation.
Rain.
Clo.
Rain
Fine
Rain
Rain
Clo.
Clo.
Fine
Fine
Clo.
Clo..
Fine
Clo.
Fine
Rain
Fine
Fine
Clo.
Fine
Fine
Fine
Fine
Fine
Fine
Fine
Rain
Fine
Fine
Fine
Fine
The quantity of rain fallen in the month of May,
is 2 inches and 90-100ths.
[ m j
Hi 1i iu ."fj -j ' <ii .') *!?? T tine; '
meteorological journal.
By Messrs. William Harris and Co. 50, Holborn, London.
Pram the 2Oth June to tlte 19/h July, 1&19, inclusive.
June
20
21
22
23
24
25
26
27
28
29
30
July
1
2
3
4
5
6
7
8
9
10
11
12
13
14
15
16
17
J8
19
o
Rain
guage
,05
fi 9
fir
,17
,27
,02
5769
58,73
6471
6568
596*
flltes 57 29-8029-70
,19
,13
o
66
681
,09 67
I'ltERM
53 30-19
6166
58 67
5961
5564
60 67
65
65
64
85
76
66
;62j68
61j65
6468
5970
64168
67:74
6568
60 65'
58*65
5765
Da rom.
30-17
:30'09
30-04
29-83
29-64
29-69
29-71
29-86
29-76
56 29 85
58 29-91
57 29'83
68 29-84
29-96
2996
30-12
30-04
30-14
30-11
30*10
29*89
29-77
29-69
2968
29*80
29-88
29*77
29-88
29-81
29-80
29-82
29-90
29-99
30*13
30 OG
30-0930*17
30-12 30-07
30-0930-02
30-1030-17
30-20 30-1
30-15 30-06
30-04'30-02
30-0230-01
30033004
29-9829-85
29-67 29-45
De Luc's
Hygrom.
Dry Damp
Wind.
N
NW
WNW
NW
sw
wsw
sw
sw
NW
WNW
VV va.
NW
wsw
WSW
wsw
w
E
NW
E
W
NNW
NW
NNW
NE
E
ENE
NE
NW
W
WSW
NNW Fine
NW
NNW
W [Fine
W
SW
Rain
RainfCIo
W
w
w
WSW
NW
NW
W
W
W
w
s
ESE
w
NNW
NW
W
NNE
Atmospheric
Variation.
Fine
Fine
Fhie
Rain
Fine
Fine
Clo.
Fine
Fine
Cfo.
Fine
Clo.
iCfo.
Cl?i.
Rain
Clo.
Fine
Fin
Fine
Cto.
?lo.
Sho.
NE Clo.
E iFine
ENE 'Clo.
E [Clo.
WNW Fine
WSW Fine
S (Fii*>
Sho.
Clo.
Rain
Fine
Fine
Fine
Fine
Fine
Fine
Clo.
Clo.
Clo.
Cfo.
CK
Rain
Clo.
Clo.
Fine
Rain
Fine
Cfo.
Sho.
Qlo.
Clo.
The quantity of rain fallen in the month of June,
is 1 inch and 67-100th?.

				

